# Pharmacokinetics of Standard- and Reduced-Dose Recombinant Human Soluble Thrombomodulin in Patients with Septic Disseminated Intravascular Coagulation during Continuous Hemodiafiltration

**DOI:** 10.3389/fmed.2017.00015

**Published:** 2017-02-21

**Authors:** Eizo Watanabe, Shingo Yamazaki, Daisuke Setoguchi, Tomohito Sadahiro, Yoshihisa Tateishi, Tatsuya Suzuki, Itsuko Ishii, Shigeto Oda

**Affiliations:** ^1^Department of Emergency and Critical Care Medicine, Graduate School of Medicine, Chiba University, Chiba City, Japan; ^2^Division of Pharmacy, Chiba University Hospital, Chiba City, Japan

**Keywords:** coagulopathy, acute kidney injury, renal dysfunction, renal replacement therapy, sepsis

## Abstract

**Introduction:**

Recombinant human soluble thrombomodulin (rTM) is reportedly excreted by the kidneys; therefore, the recommended dose for patients with renal impairment is one-third of the standard dose. The aim of this study was to evaluate whether this reduced dose of rTM achieves effective drug concentrations that are comparable to those of the standard dose in treating sepsis-induced disseminated intravascular coagulation (DIC) during continuous hemodiafiltration (CHDF).

**Methods:**

Eight patients in an intensive care unit were randomized to receive either reduced-dose (0.02 mg/kg, *n* = 4) or standard-dose (0.06 mg/kg, *n* = 4) rTM. We evaluated the effect of standard dose in comparison to that of reduced dose on the pharmacokinetics (PKs) of rTM for the sepsis-induced DIC patients receiving CHDF. Patients received rTM during a 30-min infusion for six consecutive days. PK parameters of rTM were analyzed using the one-compartment model.

**Results:**

The elimination half-life, clearance (T1/2), and distribution volume of sTM were similar between the reduced and standard doses. The maximum concentration (Cmax) and area under the concentration–time curve (AUC) of sTM were approximately 2.5 times higher with standard-dose daily infusions than that with reduced-dose drip infusions (*p* = 0.041 and 0.062, respectively). The time when the blood concentration of sTM was >500 ng/mL, i.e., the holding time, was significantly longer with standard-dose infusions than those with reduced dose (*p* = 0.039).

**Conclusion:**

rTM displayed dose-dependent PK behavior at clinically relevant doses. During CHDF, effective blood concentration of rTM was not achieved with the reduced dose, and rTM was found to not bioaccumulate. Therefore, this pilot study suggests that reducing the rTM dose is unnecessary, even in sepsis-induced DIC patients who require CHDF. However, we need to perform a definitive study to determine the dosage of rTM for the case.

## Introduction

Severe sepsis continues to be life threatening and the most common cause of death in intensive care units (ICUs) (1). Mortality from sepsis remains high; therefore, new therapies are urgently needed. In the pathophysiology of severe sepsis, disseminated intravascular coagulation (DIC) has been recently recognized to play a key role and to be a therapeutic target. However, antithrombin-III (ATIII) and tissue factor pathway inhibitor had no effect on mortality in clinical trials (2, 3). Moreover, one of the anticoagulation factors, activated protein C (APC), was promoted as an antiseptic agent (i.e., Xigris™) but was later withdrawn owing to the negative results in the PROWESS SHOCK trial (4). Currently, there is no natural anticoagulant for the treatment of sepsis-induced DIC. However, recombinant human soluble thrombomodulin (rTM, or ART-123) was reported to be a promising “sepsis drug” and was found to regulate inflammation and coagulopathy (5). rTM promotes protein C activation by thrombin, resulting in the formation of APC. Thereafter, APC inhibits thrombin generation by inactivating coagulation factors Va and VIIIa to interrupt activation of blood coagulation (6). APC also has anti-inflammatory properties, interfering especially with the activation of complement and the inactivation of high mobility group box 1 (HMGB1), a late phase death mediator in severe sepsis (7).

Recombinant human sTM has been marketed as a novel anticoagulant for DIC in Japan since 2008. Continuous hemodiafiltration (CHDF) is widely accepted as an effective modality for patients with severe sepsis as a countermeasure against hypercytokinemia and renal dysfunction (8, 9). Indeed, in a study of 29,269 critically ill patients, 47.5% of patients with acute renal failure had simultaneous septic shock (10). Despite widespread use of rTM for management of coagulopathy in patients with severe sepsis in Japan, little is known about its pharmacokinetic (PK) properties and dosing in the subset of critically ill patients experiencing coagulopathy during CHDF.

Nakashima et al. reported that mean urinary recovery of rTM within the first 48 h was between 54.3 and 59.8% of dose, which means rTM is excreted by the kidneys (11). Therefore, the recommended dose for patients with renal impairment is one-third the standard dose. A number of reports suggest that the reduced dose for patients with renal impairment is too conservative, resulting in underdosing. As underdosing is a serious problem, we investigated the PKs of standard- and reduced rTM with sepsis during continuous hemofiltration.

## Patients and Methods

### Patients

After approved by the institutional ethics committee, written informed consent was obtained from patients or their next of kin. Between September 2010 and August 2012, patients who were admitted to the mixed medical-surgical ICU of the Chiba University Hospital, Japan, were eligible for enrollment into the study, if they met the following criteria: (1) diagnosed with severe sepsis that would require CHDF as continuous renal replacement therapy and/or as a countermeasure against hypercytokinemia (8) for more than 24 h; (2) Japanese Association of Acute Medicine DIC criteria score of ≥4 (12), (3) at least 18 years of age, and (4) written informed consent was provided.

Patients were excluded if they had (1) chronic kidney diseases; (2) significant intracranial, pulmonary, or digestive hemorrhages; (3) hypersensitivity to rTM; or (4) a pregnancy or suspected pregnancy.

The diagnosis of severe sepsis was made when a patient met the criteria proposed by the American College of Chest Physicians/Society of Critical Care Medicine Consensus Conference (13).

This trial was registered at the UMIN-Clinical Trials Registry (UMIN000004074).

### Renal Replacement Therapy

Vascular access was obtained by insertion of a flexible, double-lumen catheter. The operation of the CHDF system was monitored with a personal bedside console (JUN-500/505; Junken Medical Co., Ltd., Tokyo, Japan). CHDF was performed using a polymethylmethacrylate (PMMA) membrane hemofilter with a membrane surface area of 1.0 m^2^ (Hemofeel CH-1.0N; Toray Medical Co., Ltd., Tokyo, Japan). Nafamostat mesilate (Futhan; Torii Pharmaceutical Co., Ltd., Tokyo, Japan), a synthetic protease inhibitor with anticoagulant properties, was used as an anticoagulant, with the dose adjusted to maintain an activated coagulation time of 150–170 s. The operating conditions for PMMA-CHDF were as follows: blood flow rate (QB), 80–120 mL/min; dialyzate flow rate (QD), 1 L/h with sterile bicarbonate solution and 500 mL/h; substitution flow rate (QS), usually 500 mL/h with sterile bicarbonate Ringer’s solution in the postdilution mode but controlled by the attending physician based on total water balance and hemodynamic state.

### Study Design

**Figure 1:**
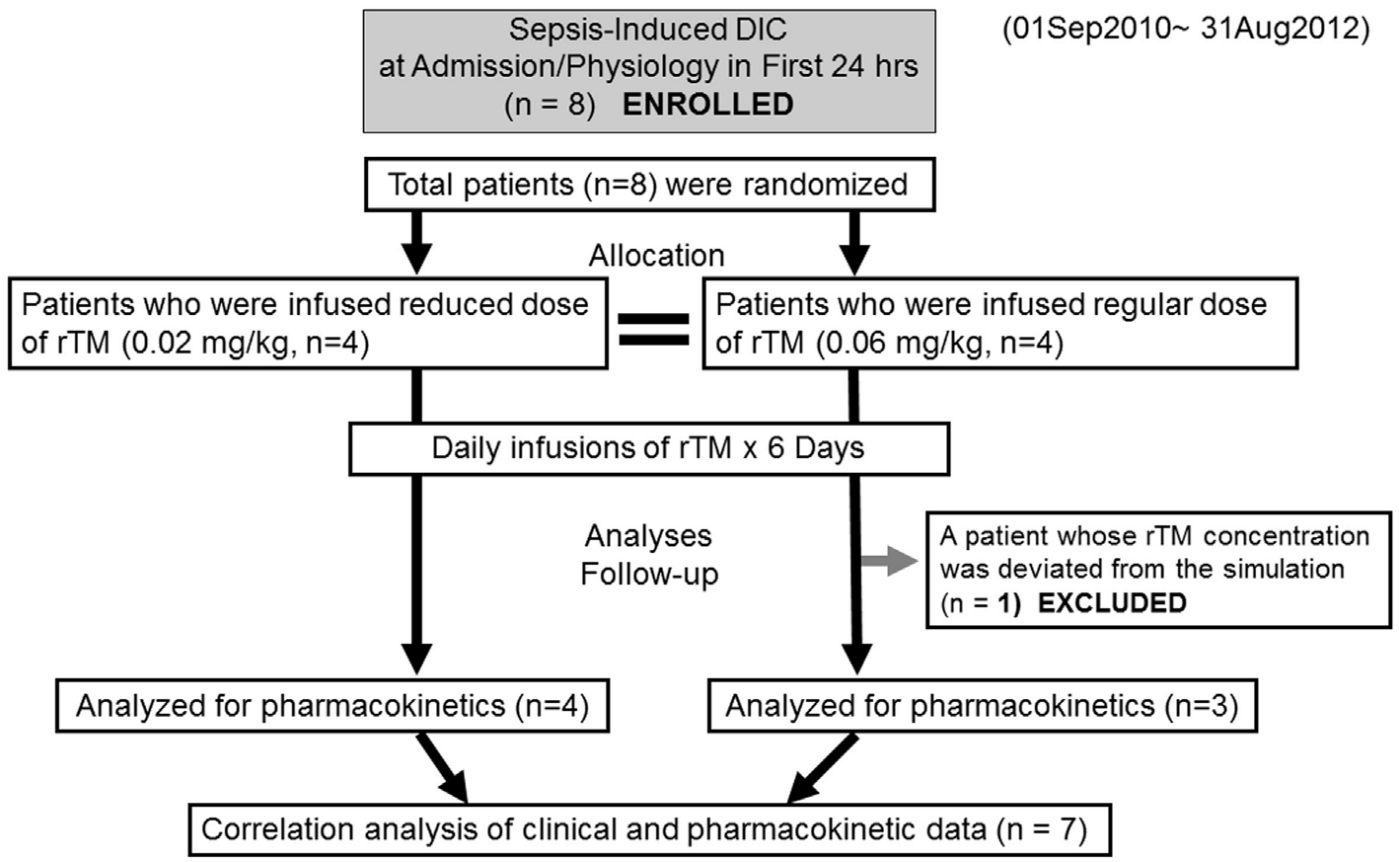
**Flow chart of patient selection disseminated intravascular coagulation and recombinant human soluble thrombomodulin**.

This study was a single-center, randomized, open, comparative design (Figure 1). The sepsis-induced DIC patients received rTM either at reduced dose (0.02 mg/kg, *n* = 4) or standard dose (0.06 mg/kg, *n* = 4) for six consecutive days. PK analyses were performed during the time of rTM administration. The sampling times were: (1) prior to the start of dosing, PD; (2) 30 min after initial administration, day 0; (3) prior to the second administration, day 1; (4) immediately after the sixth administration, day 5; and (5) 24 h after the end of the sixth administration, day 6.

This study protocol was approved by the Institutional Review Board of Chiba University Hospital in conformity with the Helsinki Protocol.

### Drug Administration and Sampling

All patients received rTM (ART-123, Recomodulin^®^; Asahi Kasei Pharma Corporation Ltd., Tokyo, Japan), which was reconstituted according to the manufacturer’s instructions. The solutions were given *via* an infusion pump over a 30-min interval. Initial sampling was done immediately after dosing completion.

All blood samples were drawn from indwelling arterial catheters into vacuum blood collection tubes containing sodium citrate. The blood was centrifuged (3,000 rpm for 10 min), and plasma separated immediately. Similarly, aliquots of dialyzate and filtrate were collected. All samples were kept at −80°C until assayed.

### Analytical Methods

The plasma rTM and soluble TM concentrations were determined with enzyme-linked immunosorbent assay (ELISA), using two different mouse monoclonal antibodies against thrombomodulin (14). The ELISA can measure the active form of TM-α because the results of ELISA correlated with those of a functional assay (15). Serum levels of interleukin-6 (IL-6) were measured daily with chemiluminescence enzyme immunoassay (CLEIA), using a rapid measurement system (Human IL-6 CLEIA, Fujirebio, Tokyo, Japan) that had been reported previously (16). HMGB1 was also measured by a commercial ELISA kit, according to manufacturer’s instructions (Shino-Test Co., Tokyo, Japan).

### PK Analysis

Because the PK of rTM can be described by a one-compartment model (14), PK parameters were determined by one-compartment analysis, using the Phoenix^®^ WinNonlin^®^ software (ver. 1.4). During the dosing period, the duration during which sTM levels were ≥500 ng/mL was recorded. We adapted 500 ng/mL as the effective concentration of sTM for holding time (the duration when the blood concentration of sTM was >500 ng/mL) because the dosage amount of rTM in clinical practice was determined by estimation from a phase 2 clinical study through all administration periods (14). Clinical test data were collected daily and correlated with the clearance of rTM (CL).

### Statistical Analysis

Values are reported as median and interquartile range, or mean ± SD (or SEM), depending on the variables. Statistically significant differences in the mean values between two groups were evaluated by the Wilcoxon signed-rank test.

Associations between the CL calculated in the present study and the clinical parameters were assessed with the Spearman’s rank correlation test.

Statistical significance was defined as a *p*-value of less than 0.05. All statistical analyses were performed using the GraphPad PRISM 5, version 5.04 (GraphPad Software, San Diego, CA, USA) for Windows.

## Results

### Patient Demographics

Eight patients were enrolled in the study and randomly allocated to two groups (reduced-dose and standard-dose administration, Figure 1). Although the cause of the sepsis-induced DIC in each group varied, age, gender, body weight, severity scores, and clinical parameters were not markedly different (Table 1). Both groups included patients with pneumonia and severe acute pancreatitis.

**Table 1:** **Characteristics of the study patients**.

	Reduced-dose rTM (0.02 mg/kg, *n* = 4)	Standard-dose rTM (0.06 mg/kg, *n* = 4)
Age (year) mean ± SD	67.8 ± 18.5	65.3 ± 17.5
Gender (male/female)	3/1	2/2
Weight (kg) median (range)	67.6 (40.5–75.7)	56.4 (49.4–58.6)
APACHE II score median (range)	22.5 (18–32)	28 (14–34)
SOFA score median (range)	13 (11–14)	12 (11–13)
SIRS score median (range)	3 (1–4)	2.5 (0–4)
JAAM DIC score median (range)	6.5 (4–8)	5.5 (5–8)
Cause of sepsis-induced DIC	• Enterocolitis	• Gas gangrene
	• Bacteremia	• Pneumocystis jiroveci pneumonia
	• Aspiration pneumonia	• Urinary tract infection
	• Bacterial translocation due to SAP	• Bacterial translocation due to SAP
sTM on admission (ng/mL) mean ± SD	9.30 ± 2.58	6.16 ± 0.65
Plt on admission (×10^4^/μL) mean ± SD	4.45 ± 0.10	5.05 ± 1.95
Creatinine (mg/dL) on admission mean ± SD	2.99 ± 1.04	1.41 ± 0.76
Cystatin C (mg/L) on admission mean ± SD	2.34 ± 0.55	1.56 ± 0.28
CLcr (mL/min) mean ± SD	22.3 ± 9.83	52.1 ± 37.3
Interleukin-6 (pg/mL) on admission Geometric mean ± SD	2214.5 ± 31.2	692.3 ± 16.3
HMGB1 on admission (ng/mL) mean ± SD	10.51 ± 6.58	10.93 ± 8.21
No. of days in ICU stay median (range)	14.5 (9–33)	20 (15–21)
28-day survival (%)	3 (75%)	3 (75%)
No. of days of CHDF median (range) (days)	7.0 (4–13)	5.5 (3–9)
DIC resolution at day 6 of rTM administration (%)	1 (25%)	2 (50%)
Bleeding-related adverse events (%)	1 (25%)	1 (25%)

*Creatinine clearance (CLcr) was calculated with Cockcroft–Gault formula*.

*SAP, severe acute pancreatitis; APACHE, acute physiology and chronic health evaluation; SOFA, sequential organ failure assessment; SIRS, systemic inflammatory response syndrome; JAAM, Japanese Association of Acute Medicine; DIC, disseminated intravascular coagulation; HMGB1, high mobility group box 1; sTM, soluble thrombomodulin; rTM, recombinant human soluble thrombomodulin; ICU, intensive care unit; CHDF, continuous hemodiafiltration; Plt, platelet count*.

Two of the eight patients (one patient in each group) died during the course of their ICU stay. In addition, two of the eight (one patient in each group) had bleeding-related adverse events during their ICU stay. One of these patients experienced bleeding because of the gastric fiberscope contacting the duodenal bulb during enteral tube insertion. Bleeding in the other patient was caused by repeated debridement for infected gas gangrene. The bleeding complications were not life threatening, none of the patients required red blood cell transfusions, and both survived (Table S1 in Supplementary Material). Therefore, any causal relationship between rTM infusion and these bleeding-related events was not defined. Although the number of patients included in the present study was less, no evaluated outcomes were different between the reduced- and standard-dose groups (Table 1).

### Pre- and Post-CHDF sTM Concentrations

**Figure 2:**
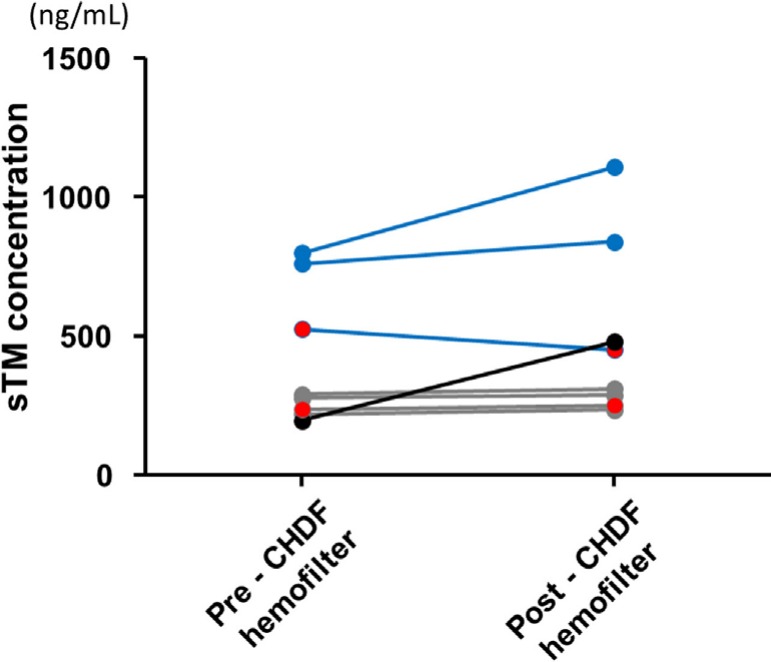
**Soluble thrombomodulin (sTM) concentration of pre- and post- continuous hemodiafiltration hemofilter right after the first infusion sTM**. The blue lines indicate the sTM concentrations of regular-dose group. The gray lines indicate the sTM concentrations of reduced-dose group. An excluded case from pharmacokinetic analyses was shown with black color which seems to show unusually low concentration of sTM at pre-CHDF hemofilter under standard-dose infusion. The two patients complicated with a hemorrhagic complication were indicated as red closed circle in each dosage group.

The sTM shed from the endothelial cell surface before administration was similar between the reduced- and standard-dose groups, and the concentrations were quite low relative to those after administration (Table 1). sTM concentrations pre- and post- CHDF hemofilter were measured on day 1, and the value in each patient was relatively elevated than that before administration (Figure 2). However, the sTM concentration of pre-CHDF plasma was statistically equivalent to that of post-CHDF plasma in both the reduced-dose and standard-dose groups (Table 2). The sTM concentrations in hemofiltrates of all patients were undetectable (i.e., below the limit of detection of 1 ng/mL). Therefore, rTM was found to be removed by neither adsorption nor filtration during CHDF.

**Table 2:** **Plasma sTM concentrations before and after CHDF after the first rTM infusion on day 1**.

	Pre-CHDF hemofilter	Post-CHDF hemofilter	*p*-Value
Reduced-dose rTM (ng/mL) (*n* = 4) median (range)	247.7 (208.2–282.6)	260.1 (223.8–302.5)	0.250
Standard-dose rTM (ng/mL) (*n* = 4) median (range)	637.4 (189.6–793.5)	653.3 (444.5–1101.8)	0.125

*CHDF, continuous hemodiafiltration; sTM, soluble thrombomodulin; rTM, recombinant human soluble thrombomodulin*.

*p-values with Wilcoxon signed-rank test*.

### PK Parameters

Table 3 shows PK parameters. The elimination half-life (T1/2), CL, and distribution volume (Vd) of rTM were similar between the reduced and standard doses (41.0 ± 7.5 vs 31.0 ± 20.9 h, 1.60 ± 0.40 vs 2.60 ± 1.20 mL/h/kg, and 91.8 ± 13.5 vs 89.3 ± 24.9 mL/kg, respectively, mean ± SD). The maximum concentration (Cmax) of rTM and the area under the curve (AUC) of rTM were approximately 2.5 times higher with standard dose than that with reduced-dose infusions (1570.9 ± 899.5 vs 615.6 ± 102.6 ng/mL, *p* = 0.041 and 140233.9 ± 91487.5 vs 57631.6 ± 9577.0 ng/mL/h, *p* = 0.062, respectively). The time when the plasma concentration of sTM was >500 ng/mL, i.e., the holding time, was significantly longer with standard dose than with reduced-dose infusions (143.3 ± 96.4 vs 33.8 ± 28.4 h, *p* = 0.039).

**Table 3:** **Pharmacokinetics of rTM**.

	Reduced-dose rTM *n* = 4	Standard-dose rTM *n* = 3	*p*-Value
T1/2 (h)	41.0 ± 7.5	31.0 ± 20.9	0.203
Cmax (ng/mL)	615.6 ± 102.6	1570.9 ± 899.5	0.041
AUC (ng⋅h/mL)	57631.6 ± 9577.0	140233.9 ± 91487.5	0.062
Vd (mL/kg)	91.8 ± 13.5	89.3 ± 24.9	0.434
CL (mL/h/kg)	1.60 ± 0.4	2.60 ± 1.2	0.113
>500 ng/mL sTM holding time, h	33.8 ± 28.4	143.3 ± 96.4	0.039

*rTM, recombinant human soluble thrombomodulin; T1/2, elimination half-life; Cmax, maximum concentration; AUC, area under the concentration–time curve; Vd, distribution volume; CL, clearance of rTM; sTM, soluble thrombomodulin*.

*p-values with unpaired t-test, one-tailed*.

*Maximum concentration (Cmax) and AUC were estimated assuming that the frequency of administration was six in each series. Values are mean ± SD*.

**Figure 3:**
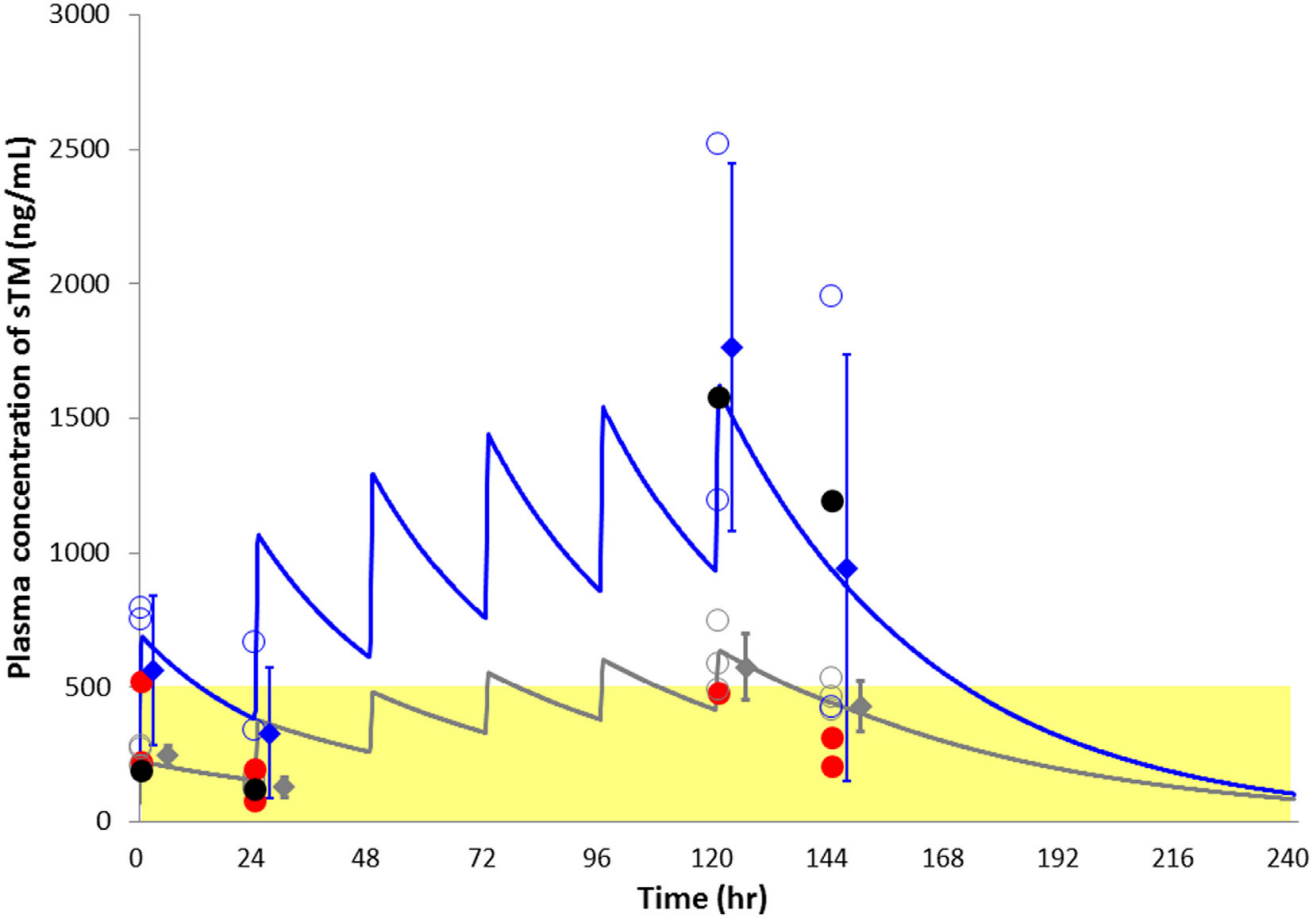
**Plasma soluble thrombomodulin (sTM) concentration with respect to time in sepsis-induced disseminated intravascular coagulation patients treated with continuous hemodiafiltration, simulated using the one-compartment model sTM**. The blue line indicates the theoretical simulating lines for sTM concentrations of standard-dose group. The gray line similarly indicates the theoretical simulating lines for sTM concentrations of reduced-dose group. The blue and gray open circles indicate actual sTM concentrations of all time points for standard- and reduced-group, respectively. An excluded case from pharmacokinetic analyses was shown with black color. The two patients complicated with a hemorrhagic complication were indicated as red closed circle in each dosage group. Data points represented as mean ± SD.

Figure 3 shows the predicted sTM concentration curves based on the mean PK parameters over 6 days. The actual concentration of sTM in one patient in the standard-dose group significantly deviated from the theoretical line (Table S2 and Figures S2A,B in Supplementary Material). Therefore, the data of this patient were excluded from the PK analyses. The sTM concentrations of patients with hemorrhagic complications (red circles in Figure 3) were relatively low even in standard-dose group.

### Correlation between CL and Clinical Parameters

**Figure 4:**
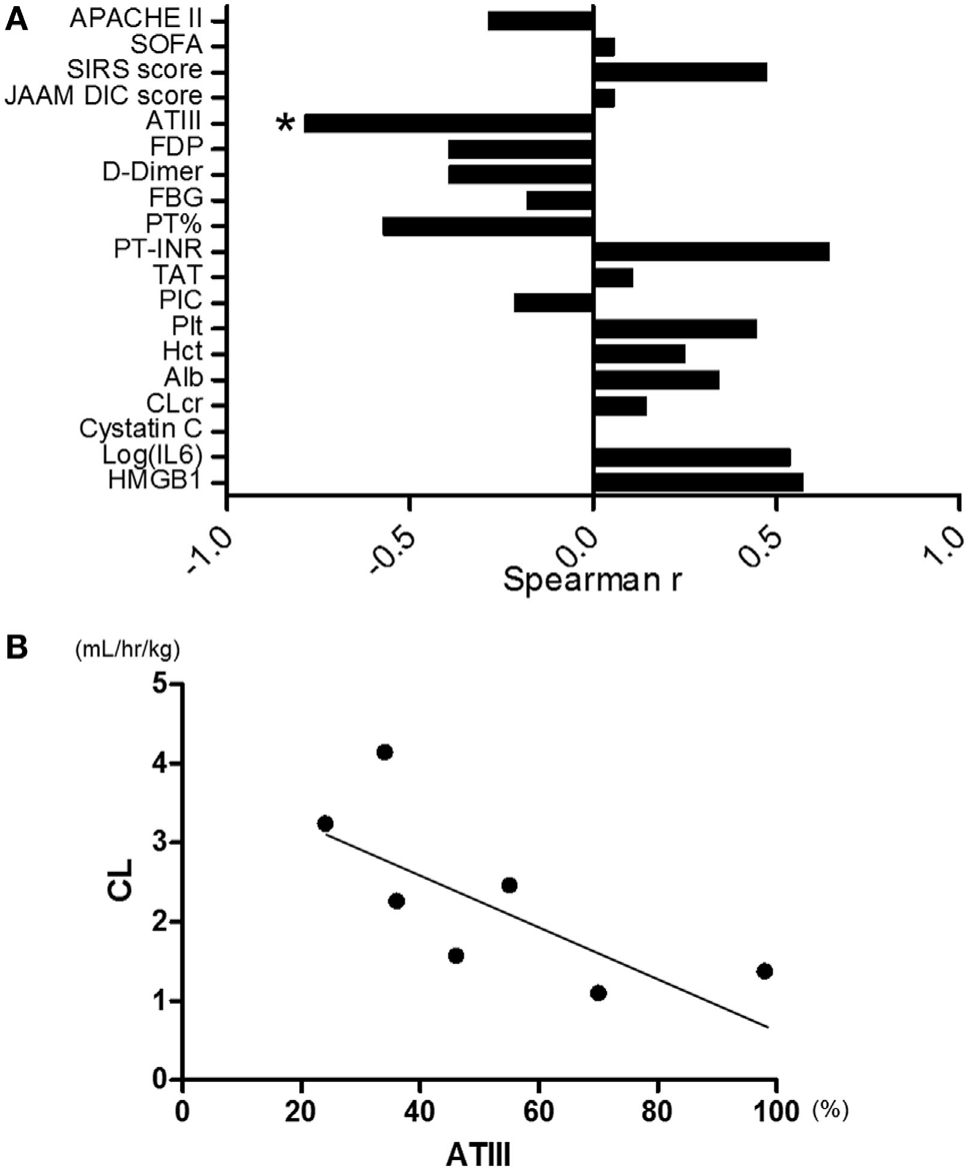
**(A)** Correlation between clearance of rTM (CL) and clinical parameters. Antithrombin-III (ATIII) activity was the only one marker that had significant correlation with CL among 20 markers evaluated (Spearman *r* = −0.821, *p* = 0.034, *n* = 7). **(B)** Correlation plots for ATIII activity relative to CL. CL was inversely correlated with ATIII activity (Spearman *r* = −0.821, *p* = 0.034, *n* = 7). JAAM, Japanese Association of Acute Medicine; DIC, disseminated intravascular coagulation; rTM, recombinant human soluble thrombomodulin. *p < 0.05 with Spearman’s rank correlation.

The correlations between CL and clinical parameters at the time of admission to the ICU were evaluated as shown in Figure 4A. Among 20 markers evaluated, ATIII activity was the only one that significantly correlated with CL (Figure 4B, Spearman *r* = −0.821, *p* = 0.034, *n* = 7). All the markers for DIC recovered toward normal value during the time course in both the reduced- and standard-dose groups (Figure S1A in Supplementary Material). Serum IL-6 levels, which are widely recognized as useful biomarkers of septic inflammation, markedly decreased during the time course. However, there was no significant difference between the two treatment groups (Figure S1B in Supplementary Material).

## Discussion

The present study is the first to demonstrate that the rTM holding time is significantly longer with standard dose than with reduced-dose infusions during CHDF. This result suggests that the efficacy of reduced-dose rTM was insufficient to regulate the coagulation system in this setting. The elevation of baseline sTM levels was also assessed after daily administration of rTM. The T1/2, CL, and Vd of rTM were similar between the reduced and standard doses, although the maximum concentration (Cmax) and the AUC of rTM were approximately 2.5 times higher with the standard dose. Therefore, rTM was found to not bioaccumulate in this setting.

Hayakawa et al. demonstrated that the CL of rTM in patients with severe renal impairment was similar to that in those without renal impairment (17). However, they analyzed the PK based on concentrations of rTM after a single infusion of the drug. We evaluated the PK of rTM using multiple sampling times (i.e., PD, day 0, 1, 2, 5, and 6) with a daily infusion regimen, which is generally recommended in the clinical setting. In contrast, Mouksassi et al. reported that a 0.06 mg/kg dose of rTM should result in drug exposure within the therapeutic range, with minimal risks of bleeding in patients with normal and impaired renal functions (18). However, the PK during CHDF was unclear prior to the present study.

Tsuruta et al. reported a formula for CL of rTM as follows: 0.00162 × WT × CLcr^0.175^ × (−0.0117 × Hct + 1) × (−0.00374 × Age + 1) (14). We compared the predicted CL using that formula with the actual CL in our patients. There was an apparent discrepancy between the actual and predicted CLs of rTM (data not shown). Creatinine clearance (CLcr) was regarded as a significant covariate for CL, which is supported by the fact that this drug is predominantly excreted by the kidney. However, CLcr did not significantly correlate with CL in our data (Figure 4A), and the CL of rTM was not affected by CHDF in the results of the present study.

In order to elucidate the mechanism of CL in renal impairment, we compared CL with the other clinical parameters by means of Spearman’s rank correlation. As shown in Figure 4A, ATIII activity most significantly correlated with CL among the 20 cofactors evaluated. Both CL and ATIII activities could be associated with the hyperpermeability of patients with severe inflammation because of the similarity in their molecular weights (i.e., 62 and 63 kDa, respectively). Leakage of both molecules to the extravascular space could cause unexplainable CL of rTM in sepsis-induced DIC patients with kidney injury. In this regard, it was reported that APC could preserve the expression of junction proteins, such as VE-cadherin for adherens junctions and occludin for tight junctions. rTM is known to promote protein C activation by thrombin, resulting in the formation of APC (19). Therefore, sufficient dosing of rTM may regulate the hyperpermeability caused by severe sepsis.

A large phase 3 study in subjects with severe sepsis and coagulopathy is currently in progress to evaluate survival benefits of rTM (http://ClinicalTrials.gov ID: NCT01598831, accessed 11 November 2016) (20). The present study should provide great impact on the future regimen of rTM. Results from a recent report suggest that dose adjustments, which are widely accepted clinically, are not necessary in patients with sepsis and DIC, regardless of renal impairment (18, 21). The present study provides novel information that dose adjustment in patients with sepsis-induced DIC during CHDF is unnecessary, despite CHDF not being able to clear rTM.

The present study has some limitations. First, the patient numbers included in this study were smaller than initially planned. After starting the present study, we decided to participate in another large-scale study, where similar patients with DIC were to be included (https://clinicaltrials.gov/ct2/show/NCT01704001?term=ART-123&rank=1; accessed on January 14, 2017). In addition, we found that the rTM holding time was significantly shorter in the reduced-dose group in preliminary analyses. Therefore, we decided to discontinue reduced-dose treatments in consideration of patient benefits and improved rTM dosage for future studies. While the number of patients included in the present study is small, these PK data are valuable because they were collected on multiple days (i.e., PD, day 0, 1, 5, and 6) from patients under such life-threatening conditions. Forty-eight sTM concentrations were included for evaluation. Furthermore, Cmax and holding time were found to be significantly different between the reduced-dose and standard-dose groups, even with the small number of patients. Second, neither clinical outcome nor complications were evaluated because of the heterogeneous patient characteristics, which are typical in ICU patient cohorts. Again, our focus was simply to study the PK of rTM during CHDF for sepsis-induced DIC. Third, the only hemofilter that we used in this study was composed of PMMA. In general, PMMA membranes have stronger adsorbing properties than other types of membrane (8). Intriguingly, despite these strong adsorbing properties of PMMA, the concentration of rTM was higher in the hemofilter outlet than in the inlet because adsorption was minimal (Table 2). This means that the hemofilter could not remove rTM during CHDF through adsorption.

Currently, anticoagulation therapy for sepsis-induced coagulopathy is recognized as a useful therapeutic strategy that is highly relevant in a small percentage of septic patients (22), although this is not discussed by the Surviving Sepsis Campaign (23). As a practical matter, the proper use of rTM, including target illness severity and dosage, as evaluated in the present study for sepsis-induced DIC patients during CHDF, is still obscure. This is true even in Japan, where rTM has already gained greater acceptance in the marketplace. Due to the anticoagulant properties of rTM, the most important concern in treating DIC patients, who are susceptible to bleeding, is a serious hemorrhage due to abrupt increases in plasma concentration of this drug. Exacerbating this concern, it is usually necessary for patients who receive CHDF to be infused with additional anticoagulant (e.g., nafamostat mesilate). The highest concentration of rTM with no bleeding event in the non-clinical toxicology studies was 5400 ng/mL, which was originally carried out in monkeys (18). Also, Shirae et al. recently reported that the highest individual simulated Cmax of the patients who received regular dose of rTM was 4730 ng/mL even though they included patients with severe renal impairment on hemodialysis in their study population (21). As for the therapeutic range, it is regarded the trough level of rTM as of >500 ng/mL on the strength of the result of phase 2 study, although there is no published report evaluating the dose effectiveness in a clinical study (14). Therefore, the baseline level of rTM is regarded as >500 ng/mL in clinical settings, although there is no report evaluating the dose effectiveness in a clinical study. Therefore, a larger cohort study that includes sepsis-induced DIC patients of varying severity is warranted to validate the necessity of dose titration (18).

## Conclusion

Recombinant human soluble thrombomodulin displayed dose-dependent PK behavior with the range of doses used in clinical settings. Effective blood concentration of rTM was not achieved with reduced-dose administration during CHDF; rTM was found to not bioaccumulate in this setting. Therefore, it might not be necessary to reduce the dose of rTM, even if sepsis-induced DIC patients are on CHDF. A prospective comparison of the clinical efficacy of the two-dose regimens using a larger patient cohort is warranted.

## Author Contributions

Conceived and designed the experiments: EW and SO. Performed the experiments: DS. Analyzed the data: SY, TSuzuki, II, and EW. Contributed reagents/materials/analysis tools: TSadahiro and YT. Wrote the paper: EW.

## Conflict of Interest Statement

The present study was partly funded by Asahi Kasei Pharma Corporation. However, this had no influence on the results of this study. The reviewer DL and handling editor declared their shared affiliation, and the handling editor states that the process nevertheless met the standards of a fair and objective review.
