# Supplementary material for: Pharmacokinetics of Standard- and Reduced-Dose Recombinant Human Soluble Thrombomodulin in Patients with Septic Disseminated Intravascular Coagulation during Continuous Hemodiafiltration
Source: Front Med (Lausanne). 2017 Feb 21;4:15. doi: 10.3389/fmed.2017.00015 (PMC5318446; doi:10.3389/fmed.2017.00015)
Supplement: Supplementary file 1 [file Data_Sheet_1.zip › Data Sheet 2.PDF]

**Supplemental Contents**

**[Supplementary Methods]**

The plasma rTM and soluble TM concentrations were determined by means of enzyme-linked immunosorbent assay (ELISA) using 2 types of mouse monoclonal antibodies against thrombomodulin (1). ELISA is able to measure the unchanged form of TM-α, because the results of ELISA correlated with those of a functional assay (2). Serum levels of interleukin-6 (IL-6) were measured with chemiluminescence enzyme immunoassay (CLEIA) daily using the rapid measurement system (Human IL-6 CLEIA, Fujirebio, Tokyo, Japan), which had been reported previously (3). HMGB1 was also measured by means of ELISA method. A commercial kit was used according to manufacturer's instruction (Shino-Test Co., Tokyo, Japan).Statistically significant differences in the mean values between the two subjects were evaluated by the Mann-Whitney *U*-test. The mean values that had time-dependent changes were analyzed by repeated measures ANOVA (post-hoc test with Bonferroni) as described in the figure legends.

**[Supplementary Results]**

Serial changes of clinical and laboratory parameters

Serial changes of clinical parameters including laboratory test findings in the two groups were evaluated as shown in Figure A.1. In all the markers for DIC were normalized after initiation of rTM during their time course in both the reduced and regular dose groups (Figure A.1a). Although there existed significant differences in basal level of both creatinine and cystatin C between the two groups ($p$=0.025 and 0.023, respectively), the time-dependent changes were similar in both the reduced and regular dose groups (Figure A.1b). Serum interleukin-6 level, which is widely recognized as one of the useful biomarkers of septic inflammation, was markedly decreased during their time courses, however there was no significant difference between the levels of those two groups. Interestingly, HMGB1 levels did not change over time in the both groups (Figure A.1b).

**[Supplementary figure legends]**

Serial changes of clinical and laboratory parameters

Figure A1a. Serial changes of DIC parameters

Results are shown as mean ± SEM (standard error of the mean) of four experiments. FDP; fibrinogen degradation products, TAT; thrombin-antithrombin complex, PIC; plasmin-α2 plasmin inhibitor complex, PT-INR; prothrombin time-international normalized ratio, DIC; disseminated intravascular coagulation. PD; prior to the start of dosing of recombinant human soluble thrombomodulin. These two curves are not significantly different from each other in all the parameters with two way repeated measures ANOVA.

Figure A1b. Serial changes of clinical parameters in relation to inflammation and renal function

Results are shown as mean ± SEM (standard error of the mean) of four experiments. HMGB1; high mobility group box 1. PD; prior to the start of dosing of recombinant human soluble thrombomodulin. These two curves are not significantly different from each other in all the parameters with two way repeated measures ANOVA.

Figure A2a. Plasma time-concentration curve simulation of sTM in case 2

sTM; soluble thrombomodulin. The blue line indicates the theoretical simulating lines for sTM concentrations of case 2. This curve simulation shows a typical line during rTM administration for sepsis-induced DIC patients on CHDF (Figure 2). The actual sTM concentration plots were exactly on the curve.

Figure A2b. Plasma time-concentration curve simulation of sTM in case 8 (the excluded case)

The blue line indicates the theoretical simulating lines for sTM concentrations of case 8. This curve simulation is deviated away from a typical line during rTM administration for sepsis-induced DIC patients on CHDF. The actual sTM concentration plots were NOT on the curve.

**[Supplementary table legends]**

Table A1. 8 patients with sepsis-induced DIC who were introduced CHDF

*Case 8 was excluded from PK analyses because actual concentration of sTM of the case was deviated from the theoretical line. GBS; Group B *Streptococcus*, MSSA; Methicillin-sensitive *Staphylococcus aureus*, MRSA: Methicillin-resistant *Staphylococcus aureus*, *E. coli*; *Escherichia coli*, DIC: disseminated intravascular coagulation, MOF; multiple organ failure

Table A2. Pharmacokinetic parameters in the 8 cases

*Case 8 was excluded from PK analyses because actual concentration of sTM of the case was deviated from the theoretical line. Elimination constant of case 8 was extremely low and the CV% was exceptionally high. Therefore, it is considered that the estimation accuracy of the case 8 is too low to include the PK data into the present analysis.

1. Tsuruta K, Yamada Y, Serada M, Tanigawara Y. Model-based analysis of covariate effects on population pharmacokinetics of thrombomodulin alfa in patients with disseminated intravascular coagulation and normal subjects. J Clin Pharmacol. 2011;51(9):1276-85.
2. Moll S, Lindley C, Pescatore S, Morrison D, Tsuruta K, Mohri M, et al. Phase I study of a novel recombinant human soluble thrombomodulin, ART-123. Journal of thrombosis and haemostasis : JTH. 2004;2(10):1745-51.
3. Matsuda K, Hirasawa H, Oda S, Shiga H, Nakanishi K. Current topics on cytokine removal technologies. Ther Apher. 2001;5(4):306-14.
