# Supplementary figures and images for: Pharmacokinetics of Standard- and Reduced-Dose Recombinant Human Soluble Thrombomodulin in Patients with Septic Disseminated Intravascular Coagulation during Continuous Hemodiafiltration
Source: Front Med (Lausanne). 2017 Feb 21;4:15. doi: 10.3389/fmed.2017.00015 (PMC5318446; doi:10.3389/fmed.2017.00015)

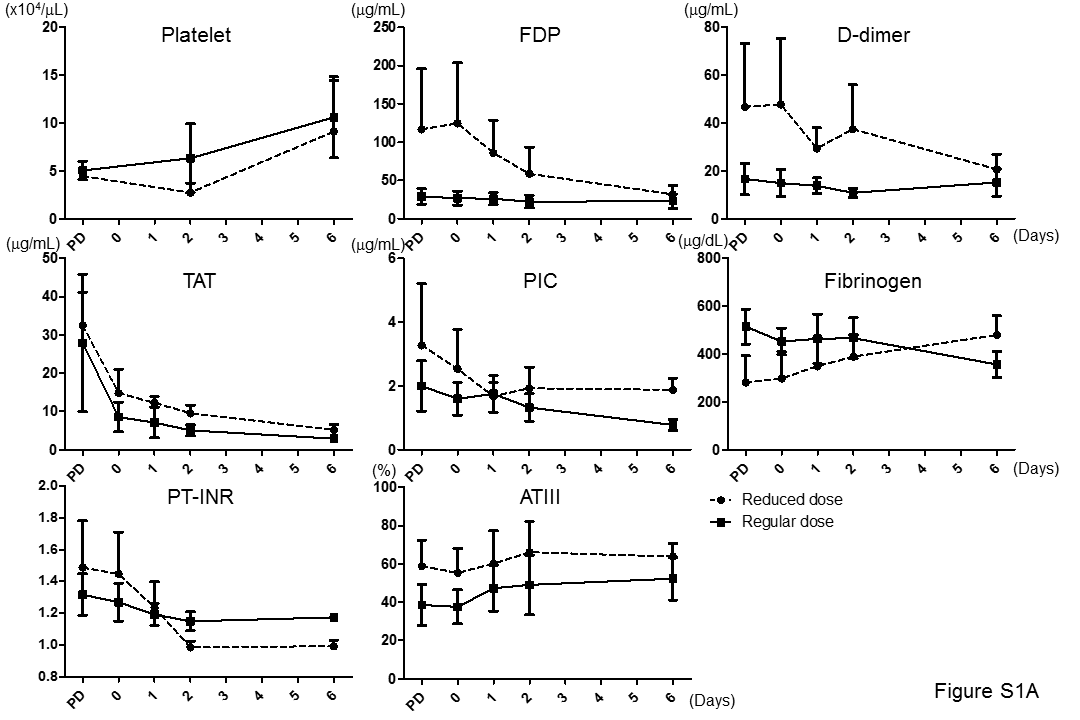

Supplement: Supplementary file 1 [file Data_Sheet_1.zip › Figure S1a.tif]

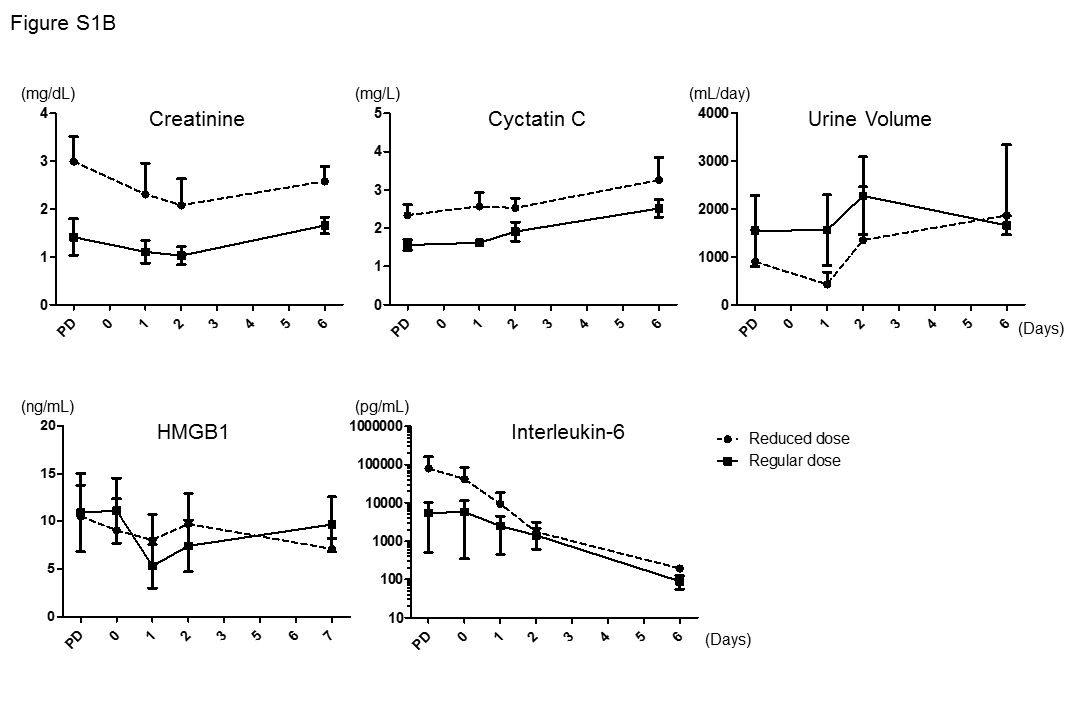

Supplement: Supplementary file 1 [file Data_Sheet_1.zip › Figure S1b.tif]

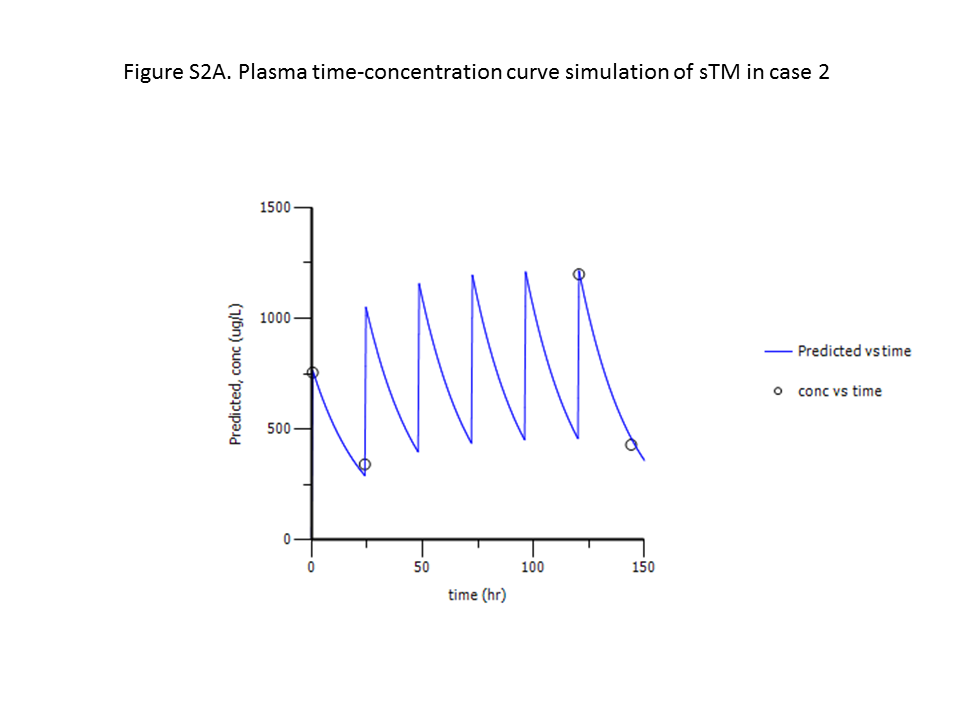

Supplement: Supplementary file 1 [file Data_Sheet_1.zip › Figure S2a.tif]

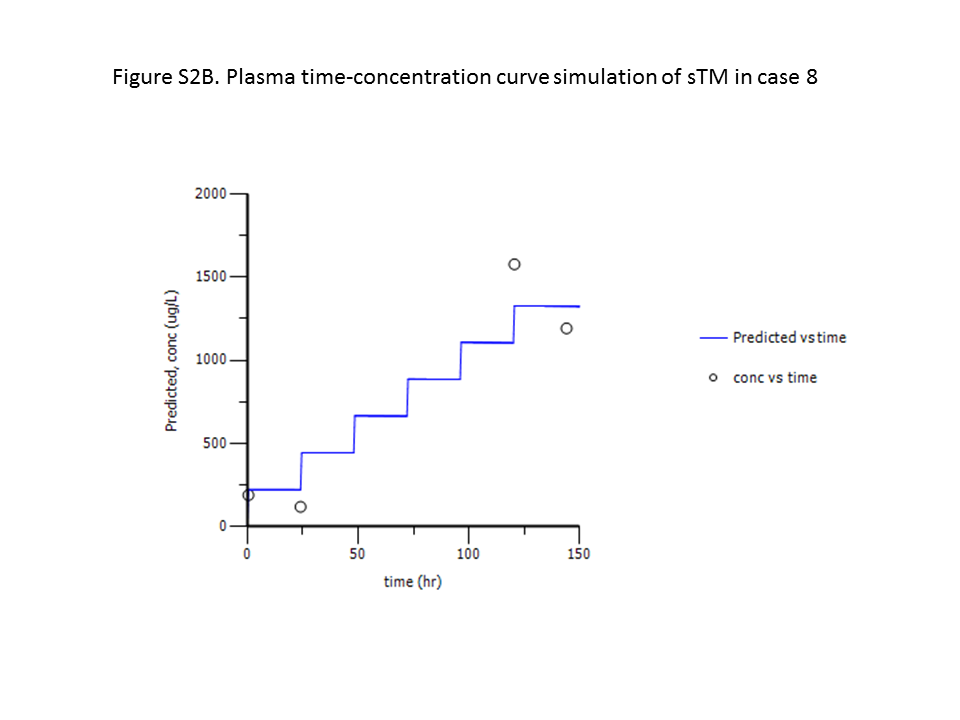

Supplement: Supplementary file 1 [file Data_Sheet_1.zip › Figure S2b.tif]

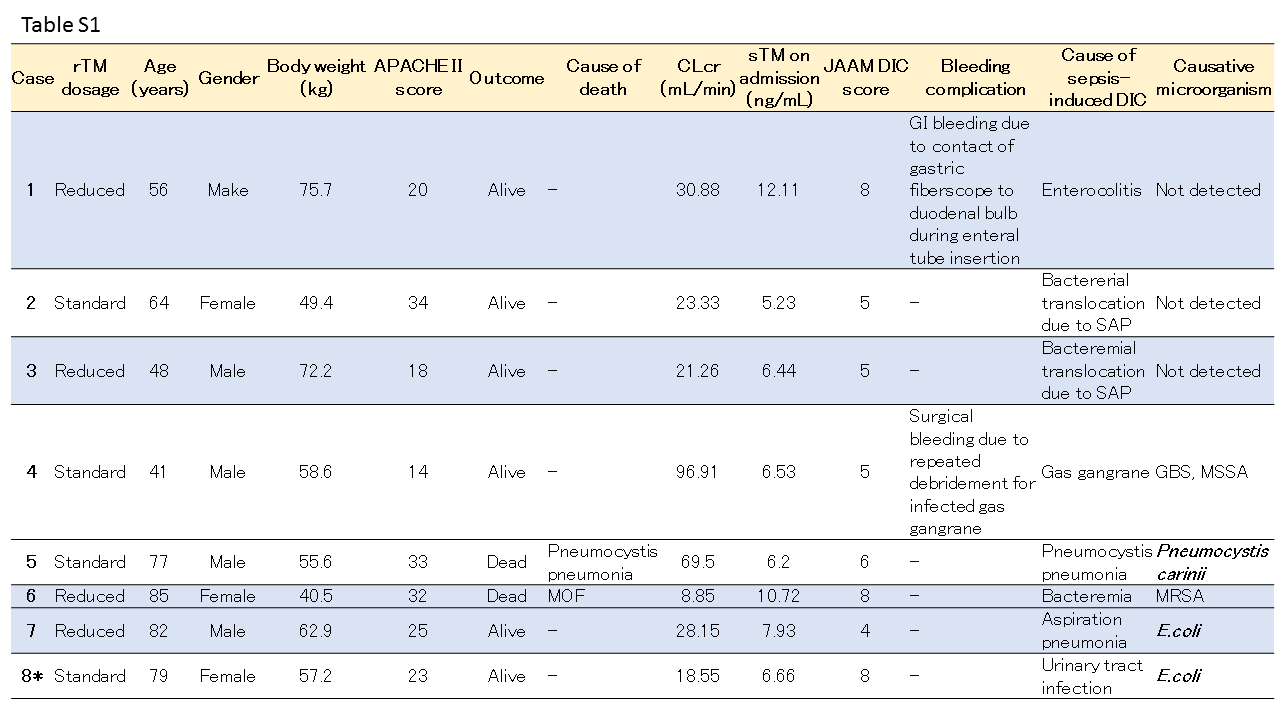

Supplement: Supplementary file 1 [file Data_Sheet_1.zip › Table S1.tif]

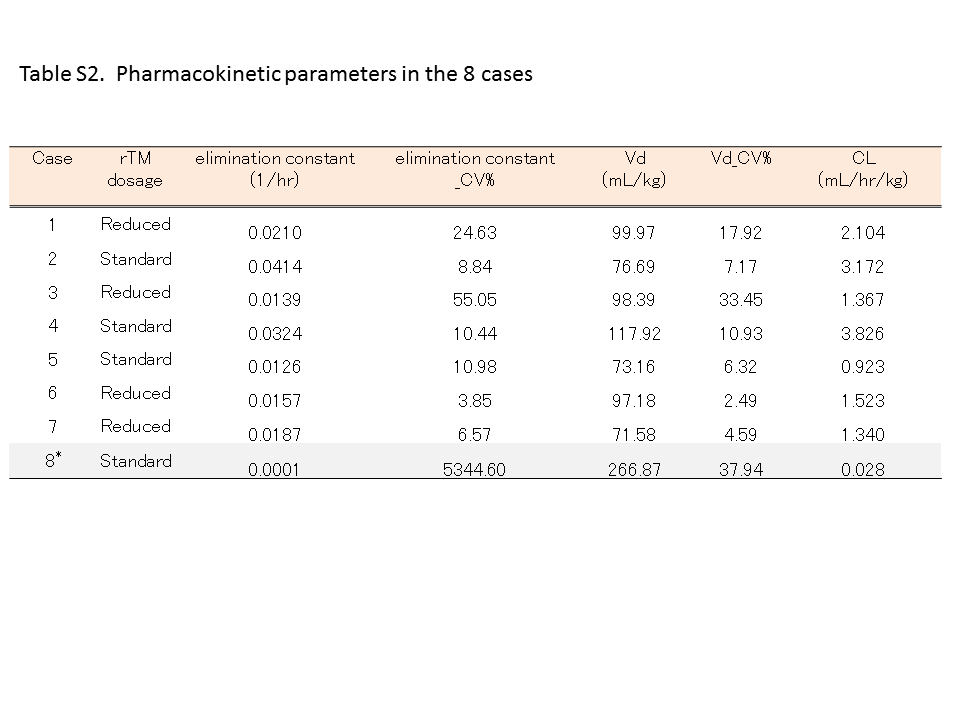

Supplement: Supplementary file 1 [file Data_Sheet_1.zip › Table S2.tif]
